# Psychometric Properties of the Proxy-Reported Life-Space Assessment in Institutionalized Settings (LSA-IS-Proxy) for Older Persons with and without Cognitive Impairment

**DOI:** 10.3390/ijerph18083872

**Published:** 2021-04-07

**Authors:** Klaus Hauer, Phoebe Ullrich, Patrick Heldmann, Laura Bauknecht, Saskia Hummel, Bastian Abel, Juergen M. Bauer, Sarah E. Lamb, Christian Werner

**Affiliations:** 1 Agaplesion Bethanien Hospital Heidelberg/Geriatric Center at the Heidelberg University, and Center of Geriatric Medicine, Heidelberg University, 69126 Heidelberg, Germany; phoebe.koepp@bethanien-heidelberg.de (P.U.); bastian.abel@bethanien-heidelberg.de (B.A.); juergen.bauer@bethanien-heidelberg.de (J.M.B.); christian.werner@bethanien-heidelberg.de (C.W.); 2 Network Aging Research (NAR), Heidelberg University, 69115 Heidelberg, Germany; heldmann@nar.uni-heidelberg.de; 3 Medical Faculty, Heidelberg University, 69120 Heidelberg, Germany; laura.bauknecht@bethanien-heidelberg.de (L.B.); saskiahum@gmail.com (S.H.); 4 Institute of Health Research, University of Exeter, South Cloisters, St. Luke’s Campus, Exeter EX1 2LU, UK; s.e.lamb@exeter.ac.uk

**Keywords:** clinical trial methods, cognitive impairment, life-space mobility, assessment, exercise, physical activity, validation, hospitalization

## Abstract

(1) Background: Life-space mobility assessments for institutionalized settings are scarce and there is a lack of comprehensive validation and focus on persons with cognitive impairment (CI). This study aims to evaluate the psychometric properties of the Life-Space Assessment for Institutionalized Settings by proxy informants (LSA-IS-proxy) for institutionalized, older persons, with and without CI. (2) Methods: Concurrent validity against the self-reported version of the LSA-IS, construct validity with established construct variables, test-retest reliability, sensitivity to change during early multidisciplinary geriatric rehabilitation treatment, and feasibility (completion rate, floor/ceiling effects) of the LSA-IS-proxy, were assessed in 94 hospitalized geriatric patients (83.3 ± 6.1 years), with and without CI. (3) Results: The LSA-IS-proxy total score showed good-to-excellent agreement with the self-reported LSA-IS (Intraclass Correlations Coefficient, ICC_3,1_ = 0.77), predominantly expected small-to-high correlations with construct variables (*r* = 0.21–0.59), good test–retest reliability (ICC_3,1_ = 0.74), significant sensitivity to change over the treatment period (18.5 ± 7.9 days; *p* < 0.001, standardized response mean = 0.44), and excellent completion rates (100%) with no floor/ceiling effects. These results were predominantly confirmed for the sub-scores of the LSA-IS-proxy and were comparable between the sub-groups with different cognitive status. (4) Conclusions: The LSA-IS-proxy has proven to be feasible, valid, reliable, and sensitive to change in hospitalized, geriatric patients with and without CI.

## 1. Introduction

The assessment of life-space mobility (LSM) is used to document an individual’s mobility in the environment considering contextual factors [1,2]. As LSM captures the habitual mobility range, it also reflects functional, environmental, and social factors that affect mobility. Independent mobility represents a prerequisite to master challenges in everyday life [3], quality of life [4], and participation in society and the natural environment [5,6]. However, during hospitalization or institutionalization, older patients have a highly sedentary behavior and spend most of their time in a lying or sitting position [7,8], with negative consequences such as a higher risk of decline in activities of daily living, new institutionalization, or death [9], underlining the need for assessment of mobility and interventions to reduce the risk of adverse outcomes. Since the concept of LSM was introduced in 1985 by May [10], it is increasingly being used to characterize the status or time course of LSM impairment, or to initiate, adjust and evaluate therapies or interventions. Several instruments have been developed to measure LSM in older adults [11]; however, with a focus on community-dwelling older persons [12].

While the mobility of community-dwelling older persons and the mobility of older persons in institutions (e.g., hospitals, rehabilitation clinics, or nursing homes) have some similarities (both depend on the physical, cognitive, psychosocial, and personal capacities of an individual [1]), they also have major differences. Life-space is commonly structured into different life-space zones within and around the home and typically includes areas such as the garden, neighborhood, hometown, etc. However, these areas are not transferable to institutionalized persons, as the type of mobility area differs substantially (home vs. ward and common areas, garden, neighborhood vs. hospital, or nursing home outdoor area). Additionally, during hospitalization, aspects related to medical status such as the severity of disease or comorbidity, to treatment such as prescribed bed-rest or medical appliances, to attitudes such as expectations towards mobility or hospital stay, and institutional aspects such as nursing to patient ratio, or availability of equipment influence individuals’ mobility behavior [13]. Many of these limitations also occur in nursing home settings [8]. The organizational structure and routines of these institutions have shown an overwhelming influence on LSM in nursing home residents [14].

Some of the factors that lead to institutionalization in hospitals or nursing homes [15,16] and negatively influence mobility behavior in older, institutionalized persons also affect or restrict the validity of self-report assessments. Accuracy of reports on health-related status in hospitalized or institutionalized persons can severely be limited by chronic conditions such as cognitive impairment (CI), which is highly prevalent in this population [17], or acute medical conditions related to critical illness such as delirium, catastrophic trauma, or exhaustion/fatigue [18,19,20]. To generate representative data, exclusion of patients with such problems in self-reporting health status may cause a systematic bias.

Currently, with the self-report Life-Space Assessment in Institutionalized Settings (LSA-IS) [21] and the Nursing Home Life-Space Diameter (NHLSD) [22], there are only two LSM surveys published and validated in institutionalized persons. The LSA-IS as an interview-based, self-report version was developed and comprehensively validated for use in hospitalized or institutionalized persons with and without CI [21]. Despite the excellent psychometric properties of the LSA-IS, the success of the assessment substantially depends on the ability of a person to be interviewed and the competence of the interviewer using the advanced interview support of the measure, which may not be provided in all settings. While collaborative, structured approaches allow assessment of the self-perspective of geriatric patients with cognitive impairment [23], such approaches have shown to be time-consuming, to require specific expertise of the assessor, and/or to have limits concerning severely cognitively impaired persons [23,24,25].

The NHLSD as an alternative and established LSM survey for institutionalized persons is based on a proxy report by the institution’s staff [22]. However, some characteristics of the NHLSD limit its applicability and some validation issues have not been addressed. First, the long observation period of two weeks compromises its use, in the hospital settings due to the varying duration of hospitalization that often tends to be shorter than two weeks.

Second, the reliability of the NHLSD and its construct validity has been analyzed by a restricted number of construct variables (functional characteristics). However, testing of detailed feasibility aspects, construct validity based on a comprehensive framework of mobility, and sensitivity to change of the NHLSD has not yet been conducted [22]. In addition, although the NHLSD provides an option to include the level of personal support in the LSM assessment, this approach has not fully been integrated into the validation.

Proxy-based information has shown to be an adequate solution in case of the inability of a person to report their health status [26,27]. However, attempts to compare patients’ and proxies’ therapy preferences or ratings on health-related status have also shown limitations of proxy reports [28,29,30], indicating problems with the congruence of self-and proxy-report. The quality of proxy reports depend on the domain being tested and the objective of the documentation, with acceptable results for lower extremity/physical functioning [31], but also references to interpret results for physical functioning with caution [32]. Data on LSM provided by a proxy as a surrogate solution for self-report have shown loss of precision, and bias with over-or under-reporting [33]. However, this direct comparison between a proxy report and a self-report has only been conducted for the University of Alabama at Birmingham Life-Space Assessment (UAB-LSA) [34], which represents an assessment for rather higher functioning community-dwelling persons with an observation period of one month. It is, therefore, less suitable to assess trajectories of LSM in institutionalized/hospitalized, impaired persons.

Thus, the objective of the present study was to comprehensively validate a new, detailed, proxy-reported LSM assessment instrument with a short observation period (proxy-reported Life-Space Assessment in Institutionalized Settings, LSA-IS-proxy), specifically designed and adjusted for institutionalized older persons in hospitals, rehabilitation settings or nursing homes. Concurrent and construct validity, test-retest reliability, sensitivity to change, and feasibility were analyzed in hospitalized geriatric patients. Differentiated analyses were also performed for subgroups according to cognitive status.

## 2. Materials and Methods

This comprehensive validation study was performed within a prospective, longitudinal cohort study to document and analyze physical activity behavior and mobility during hospitalization in geriatric patients, with and without CI (“Physical Activity in Geriatric patients during Early Rehabilitation”, PAGER; trial registration number: DRKS00016028). The study was conducted following the Declaration of Helsinki and was approved by the ethics committee of the Medical Department of the University of Heidelberg (S-709/2018).

Persons admitted to acute medical wards of a German geriatric hospital were included according to pre-defined inclusion criteria. Inclusion criteria were: receipt of complex early geriatric rehabilitation treatment according to the German hospital payment system (German Diagnosis-Related Groups), age ≥ 65 years, Mini-Mental State Examination (MMSE [35]) score ≥ 10, no delirium (Confusion Assessment Method [36]), ability to walk at least 4 m with or without walking equipment, no terminal illness, no very severe functional, sensorial or behavioral impairments that compromised study participation or assessment, no uncontrolled infection, basic communication in the German language possible, and written informed consent by the patient or the patients’ legal representative within 72 h after admission.

### 2.1. Descriptive Measures

Sociodemographic and clinical characteristics were documented from patient charts or by standardized patient interviews at hospital admission to characterize the study sample and for analysis of construct validity. Age, gender, and multi-morbidity (number of medications) were retrieved from patient charts. Trained interviewers assessed frailty status (Clinical Frailty Scale, CFS [37]), falls in the previous year [38], pain (Present Pain Intensity scale, PPI [39,40]), cognitive status (MMSE [35]), health-related quality of life (EuroQol questionnaire, EQ-5D-3L [41]), apathetic symptoms (Apathy Evaluation Scale-Clinical version, AES-C [42,43]), and concerns about falling (Short Falls Efficacy Scale-International, 7-item version, Short-FES-I [44,45]). Motor–functional status was assessed by the Activities of Daily Living (ADL) (Barthel-Index [46]), and the Short Physical Performance Battery (SPPB [47]). Physical activity (duration of activity and gait, number of steps) over 48 h was measured with the uSense, a sensor that has been validated to assess physical activity sensor in multi-morbid, geriatric patients [48].

### 2.2. Life-Space Mobility Assessment

The LSA-IS-proxy is based on an external (proxy) report and represents a newly designed LSM assessment instrument adjusted for the institutionalized environment (hospital setting) and is consistent in content and documentation with the self-report, LSA-IS version [21]. The main difference is the type of assessment (self vs. proxy report). The LSA-IS documents mobility in the environment, divided into six concentric zones within and around an institutionalized setting (level 0: own bed, level 1: own room, level 2: within the ward, level 3: within the facility, level 4: immediate outdoor area of the facility, level 5: beyond the area of the facility). Additionally, the LSA-IS considers the frequency of activity in each zone (1: 1 × per day, 2: 2–3 × per day, 3: 4–5 × per day, 4: >5 × per day), and the level of assistance needed to be mobile in each respective zone (1: with personal support, 1.5: with equipment, 2: without any support). The observation period includes the previous day (24 h). Corresponding to the LSA-IS, different outcomes can be calculated. The total score is composed of the sum of the scores for all zones, while each zone score consists of the product of zone score, frequency score, and independence score. The LSA-IS-proxy total score has a range of 0–120. Zero indicates absolute immobility (confined to bed), and 120 points as the maximal score indicate independent mobility beyond the facility’s outdoor area six or more times a day without assistance or equipment. Additionally, three sub-scores of the LSA-IS-proxy serve as detailing outcome parameters: The maximal life-space, achieved (1) with equipment or personal assistance if needed (range 0–5), (2) the life-space achieved with equipment, if needed, but without personal assistance (range 0–5), and (3) the independent life-space, achieved without any assistance (range 0–5) [34,49]. For details of the test proceeding, see the manual and assessment form attached in the Supplementary Materials (Supplementary Material File 1).

The assessment of the proxy-based version of the LSA-IS should be administered by a person involved in the organizational and treatment routines of the setting/institution, allowing comprehensive observation during the assessment period. The observation period may focus on weekdays to document LSM during routine hospital proceedings.

### 2.3. Assessment of Measurement Properties and Assessment Procedure

Concurrent validity was determined using the proxy-based and the self-report version of the LSA-IS [21], which was assessed simultaneously at hospital admission by different assessors.

For construct validity, correlational analyses were conducted between LSA-IS-proxy scores and descriptive variables that have shown moderate to high associations with LSM in previous comparable validation studies, such as studies for the UAB-LSA [34], the Life-Space Assessment in Persons with Cognitive Impairment (LSA-CI) [49] and the NHLSD [22]. Analyses were based on data assessed at hospital admission. Selection and classification of variables were conducted following a comprehensive and well-established model for mobility by Webber [1], and included the demographic variables age and gender, as variables for health status number of medications, the PPI, and the CFS, for cognitive status the MMSE [35], for the psychosocial status the EQ-5D [41], the AES-C [42,43], and the FES-I [25], for motor-functional status the ADL Barthel Index [46] and the SPPB [47], and physical activity sensor-based measurements of activity and gait duration, and the number of steps [48].

To analyze the test-retest reliability, the LSA-IS-proxy was assessed on two consecutive days by the same trained assessor, if possible, immediately after hospital admission, in case the retest was not possible after admission (due to weekend or diagnostic procedure), test and retest were conducted before discharge.

Sensitivity to change was analyzed in all available participants that could be tested at admission and immediately before the discharge, under the assumption that the “complex early geriatric rehabilitation”—as an early, multidisciplinary, geriatric rehabilitation program established in German geriatric hospitals routines—would positively affect ward-based LSM.

The feasibility of the LSA-IS-proxy was checked for completion rate, as well as floor and ceiling effects, using the baseline LSA-IS-proxy scores. Floor and ceiling effects, defined as the proportion of respondents scoring the minimal or maximal possible score, were classified as significant if more than 15% of participants achieve the lowest or highest score [50].

Assessors were members of the study group, working in the hospital daily and were familiar with the hospital procedures/organization and study participants.

### 2.4. Subgroup Analyses

All analyses were conducted for the total group and differentiated for subgroups corresponding to their cognitive status regarding the high prevalence of CI in institutions such as hospitals or nursing homes and the potential influence of CI on self-report. Participants with MMSE scores < 24 (range: 10–23) were assigned to the group of persons with CI and participants with scores ≥ 24 (range: 24–30) were assigned to the group of persons with intact cognition [35].

### 2.5. Statistical Analysis

Descriptive measures are presented as frequencies and percentages for categorical variables and means and standard deviations (SD) or medians and ranges for continuous variables as appropriate. The concurrent validity of the LSA-IS-proxy against the self-reported version of the LSA-IS and the test-retest reliability of the LSA-IS-proxy were determined by calculating intra-class correlation coefficients (ICC_3,1_) for absolute agreement with 95% confidence intervals for the total and sub-scores. ICCs below 0.4 were rated as poor, between 0.4 and 0.75 as fair to good, and above 0.75 as excellent [51]. Additionally, a Bland–Altman plot was constructed with the between-method differences (bias) and 95% limits of agreement (LOA = mean_bias_ ± 1.96 × SD_bias_) to visualize the level of agreement between the total scores of the self-and proxy-reported LSA-IS. Correlation coefficients (Spearman’s rank-order and point-biserial correlation as appropriate) between the LSA-IS-proxy scores and demographic, health, cognitive, psychological, and motor-functional status, and physical activity were calculated to assess construct validity. Correlation coefficients (*r*) of 0.1–0.3 were considered small, between 0.3–0.5 moderate, and above 0.5 high [52]. Based on previous findings [21,34,49], we expected lower correlation coefficients of the LSA-IS-proxy scores to demographic, health, cognitive, and psychological variables, representing different domains, and higher associations with motor-functional variables and physical activity, representing a common motor domain.

Sensitivity to change was examined with standardized response means (SRMs) and paired *t*-tests. Paired *t*-tests were computed to test for significant within-group differences between baseline and post-intervention assessment. To quantify the magnitude of change, SRMs were calculated as the difference in mean change scores divided by the *SD* of the change score [53]. To interpret the value of the SRMs in terms of Cohen’s thresholds for effect sizes (trivial <0.2, small ≥0.2 <0.5, moderate ≥0.5 <0.8, and large ≥0.8) [52], SRMs were adjusted for the size of correlation coefficients between the baseline and post-intervention scores [54].

A two-sided *p*-value of <0.05 indicated statistical significance. All statistical analyses were performed using the Software IBM SPSS Statistics Version 25 for Windows (IBM Corp., Armonk, NY, USA).

## 3. Results

Out of 934 patients that were admitted to the hospital during the study period, 155 were included in the PAGER study according to the predefined inclusion criteria and 94 patients were rated with the LSA-IS-proxy assessment, as the assessment of LSM was only conducted on and for weekdays to document habitual mobility during routine hospital proceedings.

### 3.1. Sample Characteristics

The total sample included 94 multi-morbid (mean number of medications: 9.9 ± 4.0), frail (clinical frailty scale mean 5.5 ± 1.0, indicating a moderate frailty status), geriatric patients (mean age 83.3 ± 6.1 years) with mild to moderate CI (MMSE: 22.8 ± 4.8) and reduced motor status (SPPB: 4.2 ± 2.4), and with 61.6% female participants. Persons with CI were slightly older and had a lower functional and physical activity status compared to persons with intact cognition. Detailed participant characteristics are summarized in Table 1. Primary diagnoses of the participants included musculoskeletal disorders (19%), neurological disorders (15%), infections (13%), acute medical illness (11%), cardiovascular disorders (10%), gastrointestinal disorders (10%), and others (23%). The number of participants included in the different psychometric property analyses varied based on the study design (*n* = 69–94 for the total group analyses).

### 3.2. Concurrent Validity 

**Figure 1:**
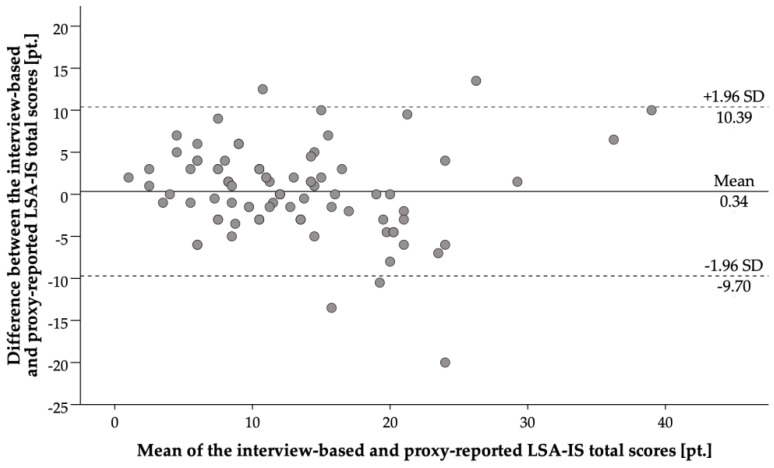
Bland-Altman plot for agreement between proxy-reported and self-report LSA-IS. Note: Presented is a Bland-Altman plot for agreement between proxy-reported and self-report LSA-IS. The solid line indicates the mean between-method difference (bias) and dashed lines the upper and lower 95% LOA (±1.96 SD of the bias).

The LSA-IS-proxy showed a good to excellent level of agreement with the self-reported LSA-IS for the total score in the total group and both subgroups (see Table 2; ICC_3,1_ = 0.69–0.79). The mean total score for the proxy-based version was 13.13 (SD 7.16), while the mean total score for the self-reported version was 12.78 (SD 5.57), with a mean difference of 0.34 (95% Confidence Interval −0.71–1.40 and LOA of −9.70 to 10.39 (Figure 1). ICCs for the sub-scores were only slightly lower, with still fair to good or excellent agreement in the total group (ICC_3,1_ = 0.59–0.70) and the subgroups (without CI: ICC_3,1_ = 0.56–0.79; with CI: ICC_3,1_ = 0.55–0.71). A Bland Altman analysis (Figure 1) displays the high association between the LSA-IS total score of the self-report vs. the proxy-reported version (see Figure 1). Overall, the level of agreement did not differ substantially between subgroups of participants according to cognitive status.

### 3.3. Construct Validity

The LSA-IS-proxy total score (total sample) showed moderate to high correlations with motor-functional status and physical activity (r = |0.30|–|0.59|), and small to moderate associations with age, frailty, cognitive status, health-related quality of life, and apathy (r = |0.21|–|0.41|), while lower and also non-significant results were obtained for gender, pain, and fall-related self-efficacy (r = |0.05|–|0.18|) as hypothesized (Table 3).

Overall, results for the LSA-IS-proxy sub-scores confirmed the results documented for the total score. For the motor-functional status and physical activity, significant correlation coefficients for the equipment-assisted life-space sub-score (r = 0.47–0.66) and predominantly significant correlation coefficients for the independent sub-score (r = 0.32–0.57) were documented. Meanwhile, the results for the maximal sub-score were lower and partially non-significant (r = 0.13–0.25). The results for demographic, health status, cognitive, and psychosocial variables varied. Significant small to high correlation coefficients were documented for the equipment-assisted sub-score and age, frailty, pain, cognition, and psychosocial variables (r = 0.27–0.56), and for the independent score and frailty (r = 0.46). Other variables of these domains showed predominantly non-significant associations with the LSA-IS sub-scores. The sub-score for equipment-assisted life-space stood out among the sub-scores with superior associations to construct variables in general.

Results for the two subgroups according to cognitive status were lower as compared to the total score, but comparable for most variables and confirming results of the total sample, with no major differences between the two sub-groups.

### 3.4. Test-Retest Reliability

Test-retest reliability was excellent to good for the total score and the equipment-assisted and independent life-space sub score, which indicates very stable results for the LSA-IS proxy in general, while the sub-score for maximal life-space showed lower reliability as compared to other scores. Subgroup analysis according to cognitive status confirmed the results for the total group and different LSA-IS scores. In trend, a slightly lower reliability occurred for the subgroup without CI (for detailed results see Table 4).

### 3.5. Sensitivity to Change

The mean duration between hospital admission and discharge and discharge was 18.5 (7.9) days (with CI: 20.1 (9.1) vs. without CI: 16.9 (6.1)). The total score and sub-scores for equipment-assisted and maximal life-space showed significant improvements over this treatment period for the total group and subgroups (single exception: maximal life-space, subgroup without CI), while for the sub-score for independent life-space no significant change could be documented. Overall, sensitivity to change was small to moderate for all LSA-IS-proxy scores (SRM = 0.32–0.58), except for the results for the independent life-space sub score (SRM = 0.07–0.13). Results for the subgroups differentiated with respect to cognitive status were comparable for all scores, with a trend for slightly higher values in the group of persons with CI (SRM = 0.17–0.58) compared to the group of persons without CI (SRM = 0.07–0.45). Results are shown in Table 5.

### 3.6. Feasibility

No missing values were documented and 100% of assessments performed in both subgroups were successful. Results for the LSA-IS-proxy total score ranged from 2 to 44, out of a range from 0 to 120 score points, indicating no ceiling or floor effects. The distribution of the LSA-IS-proxy total score was skewed, with a median score of 12 points, suggesting a highly restricted LSM as expected in the multi-morbid, vulnerable sample in the hospital setting. The range of results differed between subgroups. In persons without CI a range of 3 to 44 scores, in persons with CI, a range of 2 to 26 was documented.

None of the participants reached the maximal life-space zone without equipment or personal assistance, and only 2.1% achieved life-space level 5 with assistance by other persons, indicating no ceiling effects for the sub-scores. The lowest possible score 0 was reached by 0% for the maximal sub-score, 20.2% for the equipment-assisted sub-score, and 77.7% for the independent sub-score, indicating floor effects for the equipment-assisted and independent LSA-IS-proxy sub-scores with more than 15% of participants achieving the lowest possible score. 

Feasibility for subgroups according to cognitive status was similar for most variables except for floor effects in the equipment-assisted sub-score which were more apparent in persons with (30.4%) than without CI (10.4%). Floor effects for the independent sub-score were comparable in both subgroups (with CI: 77.1%, without CI: 78.3%). Overall, the results for the subgroups confirm the excellent feasibility results as achieved in the total group.

## 4. Discussion

Results of the present study documented an overall good concurrent and construct validity, test-retest-reliability, sensitivity to change, and feasibility of the newly developed proxy-based version of the LSA-IS. Results for LSA-IS-proxy sub-scores were in line with most results of the total score. Results for subsamples according to cognitive status were comparable for most included variables or different biometrical properties. The high completion rate confirms excellent feasibility for application in research or clinical routines.

### 4.1. Concurrent Validity

For this study, the analysis of concurrent validity was included, targeting a psychometric property not analyzed before for LSM assessments in institutionalized persons by comparing the proxy-based with the self-reported version for the same persons, period, and the setting within the same study. The only study analyzing concurrent validity of a proxy-based against a self-reported version of an LSM assessment also reported an excellent level of agreement for the UAB-LSA. However, study participants were healthy, ambulatory community-dwelling elderly without acute illness, and the analysis was only based on the UAB-LSA total score [33], limiting the comparability. Another study compared the NHLDS with a home-based Life-Space Assessment with good concurrent validity, with both tools using proxy reporting [55]. The on-average good absolute agreement for the total score, as well as the sub-scores in the present study, indicated a high assessment standard for both measures. Similar results could be achieved by two optimized assessment strategies specifically tailored for the target population represented by an interview-based self-report with supportive interview strategies [21], as compared to a proxy report by an assessor engaged in daily ward routines and organization with detailed information on patients’ activities as in the present study. Overall, results did not differ substantially between subgroups of participants according to cognitive status indicating that the proxy-based and the self-report version of the LSA-IS have successfully been developed to accurately document LSM even in vulnerable persons with CI. Although a high concurrent validity could be documented, we do not suggest a mix of both assessment methods, representing a flawed methodological approach for data on physical ability [56]. As both versions did not differ substantially for results and both present with overall good validity in multi-morbid patients with and without CI, an assessment by independent methods is preferable.

### 4.2. Construct Validity

For construct validation of the LSA-IS-proxy, we used construct variables from different domains relevant for the hospital setting within a comprehensive framework of mobility [1]. Based on their relevant association to LSM as documented in previous comparable studies [21,34,48,49,57] and oriented on an established mobility framework [1], different levels of associations to included domains were hypothesized. Present results for construct validity were in line with these assumptions between construct variables and the LSA-IS-proxy, documenting good construct validity. As expected for demographic, cognitive, psychosocial, and health status, significant moderate associations could be identified. Frailty status and age stood out among these variables and are in line with previous results for associations of physical activity and aging [58,59,60]. The lower associations of non-motor domains were expected as these variables represent “distant” domains to spatial behavioral activity measures such as the LSA-IS-proxy. As hypothesized, motor-functional status and physical activity showed moderate to high associations that were significant for all construct variables out of the common motor domain. Results were in line with previous comparable validation studies of LSM assessments (UAB-LSA, LSA-CI, NHLSD) in community-dwelling [34,49,57] or institutionalized older persons [21,22]. Tinetti & Ginter showed high associations of the NHLSD with specific diseases, while the focus in the present study laid on the functional performance [22]. In another study with a new framework for LSM, aspects of psychosocial status such as aspirations and plans or the motivation to be mobile were shown to be important for LSM [61] and might also have relevance for LSM in institutions but have not been implemented in the present study. Established associations between selected construct variables might be harder to observe in an institutionalized setting as compared to the community-dwelling setting. In an institutionalized setting, the life-space and its use are much more restricted by organizational or medical/ therapeutic routines, which in general, address all persons in such a setting [13], thereby equalizing potential effects as represented by the construct variables. Documented significant associations can therefore be considered hard evidence for the construct validity of the LSA-IS-proxy.

The sub-scores on the equipment-assisted and independent life-space followed the results for the total score. In trend, lower associations were documented for the maximal life-space sub-score, especially for associations with variables from the common motor domain. The lower association may relate to the infrequency of maximal mobility in multi-morbid, high aged, geriatric patients during acute, ward-based medical care with highly standardized organizational and therapeutic routines with less individual freedom to roam the life-space available in a hospital setting.

Good results for construct validity did not differ substantially between subgroups with and without CI in most included variables, documenting good validity in persons with moderate to more advanced cognitive impairment, who represent a high percentage if not most patients in geriatric hospitals, as well as other institutionalized settings [17,62].

### 4.3. Test-Retest Reliability

We found a good to excellent test-retest reliability for the total score, as well as the equipment-assisted and independent life-space sub-score, indicating high reliability for the LSA-IS-proxy in general. Only the sub-score on the maximal life-space showed lower reliability. Results are comparable to those reported in previous studies on LSM assessments (UAB-LSA, LSA-CI) in community-dwelling older persons [34,49] and the previously published study on the self-reported versions of the LSA-IS in a geriatric hospital setting [21]. The maximal life-space sub-score was implemented in the LSA-IS for good reasons to cover activities in the patients with above-average mobility and to prevent potential ceiling effects from a methodological perspective. The lower reliability may relate to the above-discussed range of maximal life-space in in-hospital settings, which also included distant areas of hospital-based mobility, such as walks to the cafeteria or the hospital vicinity. Such events are not part of hospital routines and depend on external support by proxies. By their infrequent and random nature, they reduce test-retest reliability especially so when tested in a short period but may not document a methodological limitation of the assessment [63,64].

Results of subgroups by cognitive status were comparable in general with a trend for lower reliability in persons without CI. The marginal differences might be based on the slightly superior functional status of the cognitively intact subgroup (see Table 1), allowing a wider range of mobility with the potential of an increased day-to-day variability with a potentially detrimental effect on test-retest reliability.

### 4.4. Sensitivity to Change

Most LSA-IS proxy scores significantly increased over the relatively short treatment period, indicating a global sensitivity to change of the assessment method with an unspecific intervention for life-space changes and a well-documented risk for hospital-associated functional decline during acute medical geriatric care [56]. Most other validation studies of LSM assessment instruments did not analyze this psychometric property which is mandatory to document the effects of interventions in research or clinical practice.

The comparison to the few previously published studies is partly limited using different statistical strategies, study design, and settings. In community-dwelling persons, Baker et al. demonstrated sensitivity to change of the UAB-LSA over time within a longitudinal, observational study design using only descriptive statistics [34]. Ullrich et al. reported comparable sensitivity to change for the LSA-CI to detect intervention-induced effects of an activity promotion program by established statistical methods as used in the present study [49]. The directly comparable study by Hauer et al. documented similar sensitivity to change for the self-report version of the LSA-IS-proxy [21]. In the present study, sensitivity to change was small to moderate for the main total score and the maximal and the equipment-assisted life-space sub-scores.

The LSA-IS-proxy documented effects of a ward-based geriatric rehabilitation, which was not primarily targeted to increase in-hospital life-space but led to significant improvements to the extent of LSM based on effects of the functional training and use of technical or personal support as part of the early, multidisciplinary geriatric rehabilitation treatment.

The lowest sensitivity to change was observed for the sub-score on the independent life-space. Only a minority of higher functioning patients not dependent on external support qualified for this sub-score, contrasting on average the low health status of geriatric patients and the mandatory ambulation support of multi-morbid persons within hospitals [65], often requested by insurance regulations even in higher functioning persons. The somewhat lower sensitivity of the independent sub-score may therefore be an indicator of a small subgroup of patients with higher functional status, resulting in a smaller intervention effect and also statistical effect of the basic early rehab training, which focuses on very basic functional limitations.

In line with other psychometric properties evaluated in this study, we found no relevant differences between subgroups with and without cognitive impairment, indicating good sensitivity to change irrespective of the cognitive status. The slightly higher sensitivity to change observed for the subgroup with CI may be based on the often-documented dose-response effect of training resulting in higher training gains for persons with initially lower motor status [66,67], as present in the subgroup with CI, than for persons with higher motor status when a standardized training is applied [68,69].

### 4.5. Feasibility

Excellent feasibility of the LSA-IS-proxy was documented based on the 100% completion rate and the complete lack of missing values in a very frail, multi-morbid population during acute medical, ward-based treatment with high failure risk for assessments. The external proxy-based approach by a trained assessor involved in daily routines of the patients guaranteed the completeness and the accuracy of reporting even when including persons with mild to more advanced stages of CI and other potential limitations with respect to self-reporting (e.g., fatigue, pain, delirium, etc.). Based on the chosen strategy by proxy reporting, no relevant differences for feasibility were documented between subgroups with and without CI. The highly organized setting with restricted degrees of freedom for mobility following standardized in-hospital routines, which are easy to document, may also have further supported proxy-based documentation to achieve this extraordinary result.

The LSA-IS-proxy was tailored to the target group, resulting in no ceiling or floor effects for the main total score, which has also been documented for the NHLSD [22], indicating excellent applicability of LSM measures to document mobility status. To prevent potential ceiling effects in persons with higher functional status, the LSA-IS includes extramural life-space areas that extend beyond the institution to document the crucial clinical transition from supervised indoor to demanding outdoor activity. These marginal areas for an institutional setting have also been included by comparable questionnaires (e.g., NHLSD) [22] or sensor-based life-space assessment [14] for the same reasons. In the present study, no relevant ceiling effects occurred in all scores/sub-scores confirming results of previous validation studies of LSM assessments with similar hierarchically structured categories of life-space zones [34,49].

We found relevant floor effects in the equipment-assisted or independent life-space sub scores. Results mirror the low health status of the multi-morbid, acutely affected study population partly not being able to be active without (independent sub-score) or even with support (equipment-assisted sub-score), leading to the documented floor effects comparable to the self-reported version of the LSA-IS or the LSA-CI [21,49]. We interpret the low LSM as a highly relevant, but so far neglected marker for health status rather than a methodological shortcoming of the assessment. The trend for low LSM was more prevalent for the equipment-assisted life-space in the subgroup with CI, confirming the ability to detect parameters highly associated with low physical and cognitive function, levels of support, and quality of life, such as life-space in more affected populations as persons with CI [21,49].

### 4.6. Limitations

The present study analyzed the LSA-IS-proxy’s psychometric properties in a geriatric hospital setting. Although the LSA-IS-proxy has been developed for generic use, formally, the generalizability of study results may have to be confirmed for other settings such as ward-based rehabilitation, nursing homes, or other comparable environments. Although the LSA-IS documents LSM of a multi-morbid, sedentary population with multiple restrictions of life-space leading to floor effects in related assessments, floor effects as documented for single sub-scores may formally represent a limitation of the assessment method.

## 5. Conclusions

The LSA-IS-proxy showed on average good feasibility, validity, reliability, and sensitivity to change in a most vulnerable population of multi-morbid, hospitalized geriatric patients irrespective of their cognitive status. Although not analyzed in the present study, the overlap of a hospital, rehabilitation, and nursing home settings with respect to organizational structures and the frail, multi-morbid populations may allow the use of the assessment in these institutionalized environments. However, a future formal repetition of the validation process may further document and analyze the biometrical quality of the LSA-IS-proxy in these different settings.

The proxy-based version ideally amends the self-reported version of the LSA-IS [21] in handicapped populations with various restrictions for accuracy and feasibility of reporting. Along with the self-report version, the LSA-IS proxy assessment allows for the first time, comprehensive and detailed documentation of life-space in a hospital setting with methodological specifications based on its organizational structure and the vulnerable health status of multi-morbid patients, including persons with cognitive impairment.

## Figures and Tables

**Table 1:** Sample characteristics.

Variables	Total Sample (*n* = 94)	Persons without CI (*n* = 48)	Persons with CI (*n* = 46)
Gender (female)	58 (61.7)	31 (64.6)	27 (58.7)
Age (years)	83.3 (6.1)	82.3 (5.9)	84.4 (6.2)
Number of medications	9.9 (4.0)	9.5 (4.0)	10.2 (4.0)
CFS	5.5 (1.0)	5.2 (0.9)	5.8 (1.0)
PPI	1.4 (1.5)	1.2 (1.3)	1.6 (1.7)
MMSE	22.8 (4.8)	26.7 (1.7)	18.8 (3.4)
EQ-5D	0.65 (0.31)	0.69 (0.28)	0.58 (0.33)
AES-C	24.3 (8.6)	26.7 (7.9)	21.7 (8.7)
Short-FES-I	12 [7–28]	11 [7–25]	12 [7–28]
Falls in the previous year	1.2 (0.8)	1.1 (0.8)	1.2 (0.8)
ADL Barthel-Index	75 [5–100]	85 [25–100]	65 [5–100]
SPPB	4.2 (2.4)	4.6 (2.3)	3.8 (2.5)
Number of steps in 48 h (uSense)	2560 (3361)	3115 (3642)	1960 (2961)
LSA-IS-proxy scores			
Total score	12.7 (7.1)	14.4 (8.3)	11.0 (5.2)
Maximal life-space	2.2 (0.7)	2.3 (0.7)	2.1 (0.8)
Equipment-assisted life-space	1.7 (1.0)	2.0 (0.9)	1.4 (1.0)
Independent life-space	0.4 (0.8)	0.4 (0.8)	0.4 (0.7)

Notes: Data are presented as n (%), median [range], or mean (SD). For single variables, the size of subsamples deviates depending on feasibility. Abbreviations: ADL Barthel-Index = Activities of daily living (range 0–100, higher values indicating higher independence in activities of daily living); AES-C: Apathy Evaluation Scale-Clinical Version (range 0–40, higher values indicating lower apathy); CFS = Clinical Frailty Scale (range 1–9, higher values indicating higher frailty status); CI = cognitive impairment; EQ-5D = EuroQol questionnaire, health-related quality of life (range 0.00–1.00, higher values indicating higher quality of life status); Short-FES-I = Short Falls Efficacy Scale-International (range 7–28, higher values indicating lower falls efficacy); LSA-IS-proxy = Life-Space Assessment for Institutionalized Settings proxy report; MMSE = Mini-Mental State Examination (range 0–30, higher values indicating higher cognitive status); *n* = numbers; PPI = Present Pain Intensity Scale (range 0–5, higher values indicating higher pain); SPPB = Short Physical Performance Battery (range 0–12, higher values indicating higher physical performance).

**Table 2:** Concurrent validity of the LSA-IS-proxy with the self-reported LSA-IS.

Self-Reported LSA-IS Scores	LSA-IS-Proxy Scores			
	Total Score	Maximal Life-Space	Equipment-Assisted Life-Space	Independent Life-Space
Total score				
total group	0.77 (0.67–0.84)			
without CI	0.79 (0.66–0.88)			
with CI	0.69 (0.50–0.82)			
Maximal life-space				
total group		0.65 (0.51–0.57)		
without CI		0.56 (0.33–0.73)		
with CI		0.71 (0.52–0.83)		
Equipment-assisted life-space				
total group			0.59 (0.44–0.71)	
without CI			0.54 (0.31–0.72)	
with CI			0.55 (0.32–0.73)	
Independent life-space				
total group				0.70 (0.58–0.79)
without CI				0.79 (0.66–0.88)
with CI				0.61 (0.39–0.76)

Note: Presented are Intraclass Correlation Coefficients (ICC_3,1_) for absolute agreement between the self-and proxy-reported LSA-IS for the total group (*n* = 93) and the subgroups with (*n* = 47) and without cognitive impairment (*n* = 46). Abbreviations: LSA-IS = Life-Space Mobility Assessment for Institutionalized Settings, CI = cognitive impairment.

**Table 3:** Construct validity of the LSA-IS-proxy.

		LSA-IS-Proxy Scores			
Variables	Group	Total Score	Maximal Life-Space	Equipment-Assisted Life-Space	Independent Life-Space
*Demographic factors*					
Age	total group	−0.31 *	−0.10	−0.37 *	−0.20
	without CI	−0.32 *	−0.06	−0.21	−0.24
	with CI	−0.26	−0.10	−0.43 **	−0.16
Gender ^1^	total group	−0.05	0.02	0.05	−0.05
	without CI	−0.06	0.05	0.12	−0.20
	with CI	−0.01	0.01	−0.04	0.01
*Health status*					
No. of medication	total group	−0.13	−0.04	−0.02	−0.13
	without CI	−0.13	−0.04	0.08	0.08
	with CI	0.10	−0.01	−0.06	−0.15
CFS	total group	−0.41 **	−0.21	−0.56 **	−0.46 **
	without CI	−0.30 *	−0.22	−0.43 **	−0.32 *
	with CI	−0.44 **	−0.21	−0.60 **	−0.60 **
PPI	total group	−0.10	0.02	−0.27 *	0.12
	without CI	−0.04	−0.16	−0.21	0.21
	with CI	−0.12	0.11	−0.27	0.04
*Cognitive Status*					
MMSE	total group	0.21 *	0.13	0.30 **	−0.02
	without CI	0.22	0.13	0.13	−0.01
	with CI	−0.13	−0.14	0.09	−0.09
*Psychosocial status*					
EQ-5D	total group	0.30 **	0.22*	0.43 **	0.21
	without CI	0.16	0.30*	0.45 **	0.07
	with CI	0.34 *	0.10	0.39 **	0.29
AES-C	total group	0.23 *	0.14	0.36 **	0.13
	without CI	0.27	0.09	0.43 **	0.08
	with CI	0.07	0.13	0.14	0.21
Short-FES-I	total group	−0.18	−0.15	−0.37 **	−0.13
	without CI	−0.08	−0.21	−0.48 **	−0.03
	with CI	−0.25	−0.08	−0.31 *	−0.25
*Motor-functional status*					
ADL Barthel Index	total group	0.47 **	0.20	0.66 **	0.35 **
	without CI	0.40 **	0.25	0.61 **	0.26
	with CI	0.46 **	0.14	0.65 **	0.47 **
SPPB	total group	0.50 **	0.25 *	0.59 **	0.43 **
	without CI	0.39 **	0.21	0.51 **	0.32 *
	with CI	0.56 **	0.28	0.60 **	0.57 **
*Physical activity*					
Duration of activity	total group	0.41 **	0.13	0.55 **	0.29 **
	without CI	0.34 *	0.16	0.47 **	0.24
	with CI	0.38 *	−0.01	0.53 **	0.36 *
Duration of gait	total group	0.53 *	0.19	0.56 **	0.43 **
	without CI	0.46 **	0.24	0.52 **	0.48 **
	with CI	0.58 **	0.10	0.60 **	0.40 **
No. of steps	total group	0.30 **	0.20	0.55 **	0.41 **
	without CI	0.45 **	0.26	0.53 **	0.44 **
	with CI	0.59 **	0.10	0.54 **	0.40 **

Note: Presented are Spearman’s rank correlation coefficients, except for gender ^1^ point-biserial correlation coefficients, showing results for the total group (*n* = 93)/persons without CI (*n* = 47)/persons with CI (*n* = 46). For single variables, size of subsamples deviates depending on feasibility (*n* = 85–94 for the total group, *n* = 44–48 for the group without CI, *n* = 41–46 persons with CI). Abbreviations: AES-C = Apathy Evaluation Scale-Clinical version; CFS = Clinical Frailty Scale; EQ-5D score = EuroQol-questionnaire; LSA-IS = Life-Space Mobility Assessment for Institutionalized Settings; MMSE = Mini-Mental State Examination; PPI = Present Pain Intensity Scale; SPPB = Short Physical Performance Battery; Short-FES-I = Short Falls Efficacy Scale-International. Correlation coefficients (*r*): small (*r* = 0.1–0.3), moderate (*r* = 0.3–0.5), or high (*r* > 0.5). * *p* < 0.05. ** *p* < 0.01.

**Table 4:** Test–retest reliability of the LSA-IS-proxy.

LSA-IS-Proxy Scores	Group	Mean (SD)		ICC (95% Confidence Interval)
		Test	Retest	
Total score	total group	14.45 (8.58)	14.50 (8.74)	0.74 (0.63–0.82)
	without CI	16.32 (10.17)	15.53 (7.95)	0.68 (0.45–0.82)
	with CI	13.01 (6.90)	13.71 (9.31)	0.80 (0.67–0.88)
Maximal life-space	total group	2.31 (0.74)	2.34 (0.81)	0.44 (0.25–0.60)
	without CI	2.43 (0.73)	2.38 (0.64)	0.36 (0.04–0.61)
	with CI	2.21 (0.74)	2.31 (0.93)	0.48 (0.23–0.67)
Equipment-assisted life-space	total group	1.92 (1.04)	1.82 (1.16)	0.76 (0.65–0.83)
	without CI	2.24 (0.93)	2.03 (0.96)	0.52 (0.24–0.72)
	with CI	1.67 (1.06)	1.67 (1.28)	0.86 (0.77–0.92)
Independent life-space	total group	0.48 (0.95)	0.47 (0.91)	0.87 (0.81–0.91)
	without CI	0.57 (1.04)	0.51 (0.90)	0.83 (0.69–0.91)
	with CI	0.42 (0.87)	0.44 (0.92)	0.91 (0.84–0.95)

Note: Presented are Intraclass Correlation Coefficients for the total group (*n* = 85) and subgroups according to cognitive status (without cognitive impairment (CI) *n* = 37, with CI *n* = 48). LSA-IS = Life-Space Mobility Assessment for Institutionalized Settings. ICC = Intraclass Correlation Coefficient (<0.4 = poor, 0.4–0.74 = fair to good, >0.75 = excellent).

**Table 5:** Sensitivity to change of the LSA-IS-proxy.

LSA-IS-Proxy Scores	Group	Mean (SD)		*p*-Value	SRM
		Baseline	Post-Intervention		
Total score	total group	13.80 (7.87)	17.70 (9.40)	>0.001	0.44
	without CI	15.13 (8.90)	18.52 (8.34)	0.048	0.39
	with CI	12.78 (6.92)	17.08 (10.21)	>0.001	0.45
Maximal life-space	total group	2.17 (0.73)	2.54 (0.80)	0.001	0.47
	without CI	2.27 (0.74)	2.50 (0.73)	0.109	0.32
	with CI	2.10 (0.72)	2.56 (0.85)	0.006	0.58
Equipment-assisted life-space	total group	1.83 (0.94)	2.23 (1.09)	>0.001	0.40
	without CI	1.93 (0.98)	2.37 (0.96)	0.021	0.45
	with CI	1.74 (0.91)	2.13 (1.17)	0.002	0.35
Independent life-space	total group	0.46 (0.88)	0.59 (1.06)	0.151	0.13
	without CI	0.50 (0.97)	0.57 (0.97)	0.601	0.07
	with CI	0.44 (0.82)	0.62 (1.14)	0.164	0.17

Note: Presented are the results of effects of early ward-based complex geriatric rehabilitation on LSM for the total group (*n* = 69) and subgroups according to cognitive status (without cognitive impairment (CI) *n* = 30, with CI *n* = 39). Only participants that were tested at baseline and directly before discharge were included. Abbreviations: LSA-IS = Life-Space Mobility Assessment for Institutionalized Settings, SRM = standardized response mean.

## Data Availability

The datasets generated and/or analyzed during the current study are not publicly available as the ethical vote did not include open data access but are available from the corresponding author on reasonable request.

## Supplementary Materials

The following are available online at https://www.mdpi.com/article/10.3390/ijerph18083872/s1, Life-Space Assessment in Institutionalized Settings (proxy LSA-IS) questionnaire form, and Life-Space Assessment for Persons in institutionalized settings reported by a Proxy (LSA-IS proxy)-User Manual/instructions.

## References

[1] Webber S.C., Porter M.M., Menec V.H. (2010). Mobility in older adults: A comprehensive framework. Gerontologyist.

[2] Parker M., Baker P.S., Allman R.M. (2002). A Life-Space Approach to Functional Assessment of Mobility in the Elderly. J. Gerontol. Soc. Work.

[3] Satariano W.A., Guralnik J.M., Jackson R.J., Marottoli R.A., Phelan E.A., Prohaska T.R. (2012). Mobility and aging: New directions for public health action. Am. J. Public Health.

[4] Metz D.H. (2000). Mobility of older people and their quality of life. Transp. Policy.

[5] Barnes L.L., Wilson R.S., Bienias J.L., de Leon C.F., Kim H.J., Buchman A.S., Bennett D.A. (2007). Correlates of life space in a volunteer cohort of older adults. Exp. Aging Res..

[6] Rosso A.L., Taylor J.A., Tabb L.P., Michael Y.L. (2013). Mobility, disability, and social engagement in older adults. J. Aging Health.

[7] Brown C.J., Redden D.T., Flood K.L., Allman R.M. (2009). The underrecognized epidemic of low mobility during hospitalization of older adults. J. Am. Geriatr. Soc..

[8] den Ouden M., Bleijlevens M.H.C., Meijers J.M.M., Zwakhalen S.M.G., Braun S.M., Tan F.E.S., Hamers J.P.H. (2015). Daily (In)Activities of Nursing Home Residents in Their Wards: An Observation Study. J. Am. Med. Dir. Assoc..

[9] Brown C.J., Friedkin R.J., Inouye S.K. (2004). Prevalence and outcomes of low mobility in hospitalized older patients. J. Am. Geriatr. Soc..

[10] May D., Nayak U.S., Isaacs B. (1985). The life-space diary: A measure of mobility in old people at home. Int. Rehabil. Med..

[11] Johnson J., Rodriguez M.A. (2020). Life-Space Mobility in the Elderly: Current Perspectives. Clin. Interv. Aging.

[12] Taylor J.K., Buchan I.E., van der Veer S.N. (2019). Assessing life-space mobility for a more holistic view on wellbeing in geriatric research and clinical practice. Aging Clin. Exp. Res..

[13] Brown C.J., Williams B.R., Woodby L.L., Davis L.L., Allman R.M. (2007). Barriers to mobility during hospitalization from the perspectives of older patients and their nurses and physicians. J. Hosp. Med..

[14] Jansen C.P., Diegelmann M., Schnabel E.L., Wahl H.W., Hauer K. (2017). Life-space and movement behavior in nursing home residents: Results of a new sensor-based assessment and associated factors. BMC Geriatr..

[15] Gill T.M., Allore H.G., Holford T.R., Guo Z. (2004). Hospitalization, restricted activity, and the development of disability among older persons. JAMA.

[16] Luppa M., Luck T., Weyerer S., Konig H.H., Brahler E., Riedel-Heller S.G. (2010). Prediction of institutionalization in the elderly. A systematic review. Age Ageing.

[17] Bickel H., Hendlmeier I., Heßler J.B., Junge M.N., Leonhardt-Achilles S., Weber J., Schäufele M. (2018). The Prevalence of Dementia and Cognitive Impairment in Hospitals. Dtsch. Arztebl. Int..

[18] Kim S., Miller M.E., Lin M., Rejeski W.J., Kritchevsky S.B., Marsh A.P., Groban L. (2018). Self- vs proxy-reported mobility using the mobility assessment tool-short form in elderly preoperative patients. Eur. Rev. Aging Phys. Act. Off. J. Eur. Group Res. Elder. Phys. Act..

[19] Lukas A., Niederecker T., Günther I., Mayer B., Nikolaus T. (2013). Self- and proxy report for the assessment of pain in patients with and without cognitive impairment Experiences gained in a geriatric hospital. Z. Gerontol. Geriatr..

[20] Gerritsen D.L., Steverink N., Ooms M.E., de Vet H.C.W., Ribbe M.W. (2007). Measurement of overall quality of life in nursing homes through self-report: The role of cognitive impairment. Qual. Life Res..

[21] Hauer K., Ullrich P., Heldmann P., Hummel S., Bauer J.M., Werner C. (2020). Validation of the interview-based life-space assessment in institutionalized settings (LSA-IS) for older persons with and without cognitive impairment. BMC Geriatr..

[22] Tinetti M.E., Ginter S.F. (1990). The nursing home life-space diameter. A measure of extent and frequency of mobility among nursing home residents. J. Am. Geriatr. Soc..

[23] Dutzi I., Schwenk M., Kirchner M., Bauer J.M., Hauer K. (2019). “What would you like to achieve?” Goal-Setting in Patients with Dementia in Geriatric Rehabilitation. BMC Geriatr.

[24] Macháčová K., Vaňková H., Holmerová I., Čábelková I., Volicer L. (2018). Ratings of activities of daily living in nursing home residents: Comparison of self- and proxy ratings with actual performance and the impact of cognitive status. Eur. J. Ageing.

[25] Hauer K., Yardley L., Beyer N., Kempen G., Dias N., Campbell M., Becker C., Todd C. (2010). Validation of the Falls Efficacy Scale and Falls Efficacy Scale International in geriatric patients with and without cognitive impairment: Results of self-report and interview-based questionnaires. Gerontology.

[26] Iezzoni L.I., McCarthy E.P., Davis R.B., Siebens H. (2000). Mobility problems and perceptions of disability by self-respondents and proxy respondents. Med. Care.

[27] Lapin B.R., Thompson N.R., Schuster A., Katzan I.L. (2019). Patient versus proxy response on global health scales: No meaningful DIFference. Qual. Life Res. Int. J. Qual. Life Asp. Treat. Care Rehabil..

[28] Pfisterer M.H., Johnson T.M., Jenetzky E., Hauer K., Oster P. (2007). Geriatric patients’ preferences for treatment of urinary incontinence: A study of hospitalized, cognitively competent adults aged 80 and older. J. Am. Geriatr. Soc..

[29] Howland M., Allan K.C., Carlton C.E., Tatsuoka C., Smyth K.A., Sajatovic M. (2017). Patient-rated versus proxy-rated cognitive and functional measures in older adults. Patient Relat. Outcome Meas..

[30] Yasuda N., Zimmerman S., Hawkes W.G., Gruber-Baldini A.L., Hebel J.R., Magaziner J. (2004). Concordance of proxy-perceived change and measured change in multiple domains of function in older persons. J. Am. Geriatr. Soc..

[31] Magaziner J., Zimmerman S.I., Gruber-Baldini A.L., Hebel J.R., Fox K.M. (1997). Proxy reporting in five areas of functional status. Comparison with self-reports and observations of performance. Am. J. Epidemiol..

[32] Li M., Harris I., Lu Z.K. (2015). Differences in proxy-reported and patient-reported outcomes: Assessing health and functional status among medicare beneficiaries. BMC Med. Res. Methodol..

[33] Cavanaugh J.T., Crawford K. (2014). Life-Space Assessment and Physical Activity Scale for the Elderly: Validity of proxy informant responses. Arch. Phys. Med. Rehabil..

[34] Baker P.S., Bodner E.V., Allman R.M. (2003). Measuring life-space mobility in community-dwelling older adults. J. Am. Geriatr. Soc..

[35] Folstein M.F., Folstein S.E., McHugh P.R. (1975). “Mini-mental state”. A practical method for grading the cognitive state of patients for the clinician. J. Psychiatr. Res..

[36] Inouye S.K., van Dyck C.H., Alessi C.A., Balkin S., Siegal A.P., Horwitz R.I. (1990). Clarifying confusion: The confusion assessment method. A new method for detection of delirium. Ann. Intern. Med..

[37] Rockwood K., Song X., MacKnight C., Bergman H., Hogan D., McDowell I., Mitnitski A. (2005). A global clinical measure of fitness and frailty in elderly people. CMAJ.

[38] Hauer K., Lamb S., Jorstad E., Todd C., Becker C., PROFANE-Group (2006). Systematic review of definitions and methods of measuring falls in randomised controlled fall prevention trials. Age Ageing.

[39] Ferrell B.A., Ferrell B.R., Rivera L. (1995). Pain in cognitively impaired nursing home patients. J. Pain Symptom Manag..

[40] Schuler M., Njoo N., Hestermann M., Oster P., Hauer K. (2004). Acute and chronic pain in geriatrics: Clinical characteristics of pain and the influence of cognition. Pain Med..

[41] Rabin R., de Charro F. (2001). EQ-5D: A measure of health status from the EuroQol Group. Ann. Med..

[42] Marin R.S., Biedrzycki R.C., Firinciogullari S. (1991). Reliability and validity of the Apathy Evaluation Scale. Psychiatry Res..

[43] Lueken U., Seidl U., Völker L., Schweiger E., Kruse A., Schröder J. (2007). Development of a short version of the Apathy Evaluation Scale specifically adapted for demented nursing home residents. Am. J. Geriatr. Psychiatry Off. J. Am. Assoc. Geriatr. Psychiatry.

[44] Hauer K., Kempen G.I., Schwenk M., Yardley L., Beyer N., Todd C., Oster P., Zijlstra G.A. (2011). Validity and sensitivity to change of the falls efficacy scales international to assess fear of falling in older adults with and without cognitive impairment. Gerontology.

[45] Kempen G.I., Yardley L., van Haastregt J.C., Zijlstra G.A., Beyer N., Hauer K., Todd C. (2008). The Short FES-I: A shortened version of the falls efficacy scale-international to assess fear of falling. Age Ageing.

[46] Mahoney F.I., Barthel D.W. (1965). Functional evaluation: the barthel index. Md. State Med. J..

[47] Guralnik J.M., Simonsick E.M., Ferrucci L., Glynn R.J., Berkman L.F., Blazer D.G., Scherr P.A., Wallace R.B. (1994). A short physical performance battery assessing lower extremity function: Association with self-reported disability and prediction of mortality and nursing home admission. J. Gerontol..

[48] Bongartz M., Kiss R., Lacroix A., Eckert T., Ullrich P., Jansen C.P., Feisst M., Mellone S., Chiari L., Becker C. (2019). Validity, reliability, and feasibility of the uSense activity monitor to register physical activity and gait performance in habitual settings of geriatric patients. Physiol. Meas..

[49] Ullrich P., Werner C., Bongartz M., Kiss R., Bauer J., Hauer K. (2019). Validation of a Modified Life-Space Assessment in Multimorbid Older Persons With Cognitive Impairment. Gerontologist.

[50] McHorney C.A., Tarlov A.R. (1995). Individual-patient monitoring in clinical practice: Are available health status surveys adequate?. Qual. Life Res. Int. J. Qual. Life Asp. Treat. Care Rehabil..

[51] Fleiss J.L. (1986). The Design and Analysis of Clinical Experiments.

[52] Cohen J. (1988). Statistical Power Analysis for the Behavioral Sciences.

[53] Katz J.N., Larson M.G., Phillips C.B., Fossel A.H., Liang M.H. (1992). Comparative measurement sensitivity of short and longer health status instruments. Med. Care.

[54] Middel B., van Sonderen E. (2002). Statistical significant change versus relevant or important change in (quasi) experimental design: Some conceptual and methodological problems in estimating magnitude of intervention-related change in health services research. Int. J. Integr. Care.

[55] Tanaka S., Yamagami T. (2018). Life-space and Related Factors for the Elderly in a Geriatric Health Service Facility. Prog. Rehabil. Med..

[56] Sakshaug J.W. (2014). Proxy Reporting in Health Surveys. T.P. Johnson, Health Survey Methods.

[57] Peel C., Baker P.S., Roth D.L., Brown C.J., Bodner E.V., Allman R.M. (2005). Assessing mobility in older adults: The UAB Study of Aging Life-Space Assessment. Phys. Ther..

[58] Umstattd Meyer M.R., Janke M.C., Beaujean A.A. (2014). Predictors of older adults’ personal and community mobility: Using a comprehensive theoretical mobility framework. Gerontologist.

[59] Kuspinar A., Verschoor C.P., Beauchamp M.K., Dushoff J., Ma J., Amster E., Bassim C., Dal Bello-Haas V., Gregory M.A., Harris J.E. (2020). Modifiable factors related to life-space mobility in community-dwelling older adults: Results from the Canadian Longitudinal Study on Aging. BMC Geriatr..

[60] Ullrich P., Eckert T., Bongartz M., Werner C., Kiss R., Bauer J.M., Hauer K. (2018). Life-space mobility in older persons with cognitive impairment after discharge from geriatric rehabilitation. Arch. Gerontol. Geriatr..

[61] Seinsche J., Zijlstra W., Giannouli E. (2020). Motility in Frail Older Adults: Operationalization of a New Framework and First Insights into Its Relationship with Physical Activity and Life-Space Mobility: An Exploratory Study. Int. J. Environ. Res. Public Health.

[62] Hoffmann F., Kaduszkiewicz H., Glaeske G., van den Bussche H., Koller D. (2014). Prevalence of dementia in nursing home and community-dwelling older adults in Germany. Aging Clin. Exp. Res..

[63] Busse M.E., Tyson S.F. (2007). Functional balance and mobility tests in healthy participants: Reliability, error and influencing factors. Physiother. Res. Int. J. Res. Clin. Phys. Ther..

[64] Trautwein S., Maurus P., Barisch-Fritz B., Hadzic A., Woll A. (2019). Recommended motor assessments based on psychometric properties in individuals with dementia: A systematic review. Eur. Rev. Aging Phys. Act. Off. J. Eur. Group Res. Elder. Phys. Act..

[65] Gell N.M., Wallace R.B., LaCroix A.Z., Mroz T.M., Patel K.V. (2015). Mobility Device Use in Older Adults and Incidence of Falls and Worry About Falling: Findings from the 2011–2012 National Health and Aging Trends Study. J. Am. Geriatr. Soc..

[66] Quisenberry A.J., Snider S.E., Bickel W.K. (2016). The Return of Rate Dependence. Behav. Anal..

[67] Laughlin M.H., Joseph B. (2004). Wolfe Memorial lecture. Physical activity in prevention and treatment of coronary disease: The battle line is in exercise vascular cell biology. Med. Sci. Sports Exerc..

[68] Werner C., Rosner R., Wiloth S., Lemke N.C., Bauer J.M., Hauer K. (2018). Time course of changes in motor-cognitive exergame performances during task-specific training in patients with dementia: Identification and predictors of early training response. J. Neuroeng. Rehabil..

[69] Ullrich P., Werner C., Bongartz M., Eckert T., Abel B., Schönstein A., Kiss R., Hauer K. (2020). Increasing Life-Space Mobility in community-dwelling older persons with cognitive impairment following rehabilitation: A randomized controlled trial. J. Gerontol. Ser. A Biol. Sci. Med. Sci..

