# Supplementary material for: Psychometric Properties of the Proxy-Reported Life-Space Assessment in Institutionalized Settings (LSA-IS-Proxy) for Older Persons with and without Cognitive Impairment
Source: Int J Environ Res Public Health. 2021 Apr 7;18(8):3872. doi: 10.3390/ijerph18083872 (PMC8067867; doi:10.3390/ijerph18083872)
Supplement: Supplementary file 1 [file ijerph-18-03872-s001.pdf]

Supplementary Materials

Life-Space Assessment in Institutionalized Settings (proxy LSA-IS) and LSA-IS proxy Manual

Table S1: Life-Space Assessment in Institutionalized Settings (LSA-IS)

**Name:**			**Date:**	
**All questions refer to the patient´s activities within the previous day.**				
**Life-space level**		**Frequency**	INDEPENDENCE	**Score**
**Has the patient yesterday been active…**		**How often the patient get there?**	**Did the patient use aids or equipment? Did he/she need help from another person?**	Level x Frequency x Independence
Level 1…<br>**within his/her room?**	1 = Yes<br>0 = No	1 = 1 × /day<br>2 = 2-3 × /day<br>3 = 4-5 × /day<br>4 = >5 × /day	1 = Personal assistance<br>1.5 = Equipment only<br>2 = No equipment or personal assistance	________<br>*Level 1 scores*
Score	____ ×	_____ ×	_____ =	
Level 2…<br>**...out of his/her room but within the ward?**	2 = Yes<br>0 = N0	1 = 1 × /day<br>2 = 2-3 × /day<br>3 = 4-5 × /day<br>4 = >5 × /day	1 = Personal assistance<br>1.5 = Equipment only<br>2 = No equipment or personal assistance	________<br>*Level 2 scores*
Score	____ ×	_____ ×	_____ =	
Level 3…<br>**Out of the ward but within the institution building (other wards, cafeteria, chapel)?**	3 = Yes<br>0 = No	1 = 1 × /day<br>2 = 2-3 × /day<br>3 = 4-5 × /day<br>4 = >5 × /day	1 = Personal assistance<br>1.5 = Equipment only<br>2 = No equipment or personal assistance	________<br>*Level 3 scores*
Score	____ ×	_____ ×	_____ =	
Level 4…<br>**Out of the institution building, but within the outdoor area of the institution (garden, park)?**	4 = Yes<br>0 = No	1 = 1 × /day<br>2 = 2-3 × /day<br>3 = 4-5 × /day<br>4 = >5 × /day	1 = Personal assistance<br>1.5 = Equipment only<br>2 = No equipment or personal assistance	________<br>*Level 4 scores*
Score	____ ×	_____ ×	_____ =	
Level 5…<br>**out of the outdoor area of your institution?**	5 =Yes<br>0 = No	1 = 1 × /day<br>2 = 2-3 × /day<br>3 = 4-5 × /day<br>4 = >5 × /day	1 = Personal assistance<br>1.5 = Equipment only<br>2 = No equipment or personal assistance	________<br>*Level 5 scores*
Score	____ ×	_____ ×	_____ =	
**Total score (LSA-IS-T)**				________<br>*Sum*
Max. Level (LSA-IS-M)				______
Max. equipment-assisted Level without personal assistance (LSA-IS-E)				______
Max. independent Level without equipment and without personal support (LSA-IS-I)				______

Supplementary File 2:

Life-Space Assessment for Persons in institutionalized settings reported by a Proxy (LSA-IS proxy)

User Manual

Introduction

The proxy – based Life-Space Assessment for institutionalized settings (LSA-IS proxy) has been developed analogue to the LSA-IS (Hauer et al., 2020) on the basis of the Nursing Home Life-Space Diameter (Tinetti & Ginter, 1990), the University of Alabama at Birmingham – Life Space Assessment (Baker, Bodner, & Allman, 2003) and the Life-Space Assessment for Persons with Cognitive Impairment (Ullrich et al., 2019) to assess life-space mobility in older persons in institutionalized settings. It assesses mobility within geographically defined areas (room, ward, whole indoor facility, outdoor area of the facility, and areas beyond the area of the facility e.g. public area/neighborhood, town and beyond), including the frequency of movement and the need of support by equipment or other persons. The assessment has to be conducted by a proxy that is familiar with the patient, the procedures of the institution, and the local environment.

Target population

The target population includes older persons in institutions, such as hospitals, rehabilitation clinics, nursing homes or other care institutions. These persons might suffer from multi-morbidity, acute medical syndromes, cognitive impairment, or disorientation due to recent institutionalization/hospitalization.

Preparation for administration

No specific extended training is needed for health professionals to administrate the LSA-IS and the assessment does not require any additional material for administration other than the questionnaire and a pencil.

Administration guidelines

The LSA-IS proxy assessed life-space mobility by a proxy representative for the patient. It needs to be conducted by a person that is familiar with the patient and its capacities, familiar with the procedures in the institution (typical daily routines such as meals, standardized therapy or care, social activities such as sport group), and the local environment (garden or park available, shopping opportunity in the immediate surrounding etc.). The proxy should have the opportunity to observe the patient without interfering the patient`s mobility. The proxy should consider obvious motor impairments and the use of equipment to achieve a realistic impression of the mobility status**.**

Optionally, the proxy could write short notions during the day in an extra form to docuemtn mobility.

Preliminary Step, if possible: During the observation period, write down short remarks referring to the participant`s mobility whenever it is possible.

Step 1: Rate the mobility for each of the levels considering independence and frequency. Optionally, use your remarks to recall the mobility during the observed day.

Step 2: To improve the accuracy of the report, you should once again critically examine the observations. Keep in mind that the LSA-CI is a measure of actual mobility - what the subject has done and not what the subject might be doing - within the previous day.

Step3: Check, if the results are consistent (e.g. independence and frequency can only be lower in a higher life-space zone, you cannot skip a life-space zone).

For Information Please Contact:

Prof. Dr. Klaus Hauer

Department of Geriatrics, University of Heidelberg

phone: +49 6221 / 319-1783

email: khauer@bethanien-heidelberg.de

Agaplesion Bethanien Hospital,

Rohrbacher Str. 149,

69126 Heidelberg, Germany

References

Baker, P. S., Bodner, E. V., & Allman, R. M. (2003). Measuring life-space mobility in community-dwelling older adults. *J Am Geriatr Soc, 51*(11), 1610-1614. doi: 10.1046/j.1532-5415.2003.51512.x

Hauer, K., Ullrich, P., Heldmann, P., Hummel, S., Bauer, J. M., & Werner, C. (2020). Validation of the interview-based life-space assessment in institutionalized settings (LSA-IS) for older persons with and without cognitive impairment. *BMC Geriatr, 20*(1), 534. doi: 10.1186/s12877-020-01927-8

Tinetti, M. E., & Ginter, S. F. (1990). The nursing home life-space diameter. A measure of extent and frequency of mobility among nursing home residents. *J Am Geriatr Soc, 38*(12), 1311-1315.

Ullrich, P., Werner, C., Bongartz, M., Kiss, R., Bauer, J., & Hauer, K. (2019). Validation of a Modified Life-Space Assessment in Multimorbid Older Persons With Cognitive Impairment. *Gerontologist, 59*(2), e66-e75. doi: 10.1093/geront/gnx214
